# Measuring the effectiveness of an automated text messaging active surveillance system for COVID-19 in the south of Ireland, March to April 2020

**DOI:** 10.2807/1560-7917.ES.2020.25.23.2000972

**Published:** 2020-06-11

**Authors:** Peter M Barrett, Niamh Bambury, Louise Kelly, Rosalind Condon, Janice Crompton, Anne Sheahan, on behalf of the regional Department of Public Health

**Affiliations:** 1 Department of Public Health HSE-South (Cork and Kerry), St. Finbarr’s Hospital, Cork, Ireland; 2 Wellcome Trust-HRB Irish Clinical Academic Training (ICAT) Programme, University College Cork, Cork, Ireland; 3 Cork University Hospital, Cork, Ireland

**Keywords:** COVID-19, active surveillance, Ireland, public health, outbreak control

## Abstract

We report the effectiveness of automated text messaging for active surveillance of asymptomatic close contacts of coronavirus disease (COVID-19) cases in the Cork/Kerry region of Ireland. In the first 7 weeks of the COVID-19 outbreak, 1,336 close contacts received 12,421 automated texts. Overall, 120 contacts (9.0%) reported symptoms which required referral for testing and 35 (2.6%) tested positive for COVID-19. Non-response was high (n = 2,121; 17.1%) and this required substantial clinical and administrative resources for follow-up.

The first case of coronavirus disease (COVID-19) was notified in Ireland on 29 February 2020 [1]. The Cork/Kerry region of Ireland is the second largest public health region in the country, and has a population of ca 690,000. The first case of COVID-19 in Cork/Kerry was notified on 5 March and by 5 June 2020, there were more than 1,800 confirmed cases in the region [2].

As part of ongoing efforts to control the spread of infection, national and international guidance recommends active surveillance of asymptomatic close contacts of confirmed cases of COVID-19 [3-7]. However, evidence for the effectiveness of active surveillance systems among community-based close contacts of cases of COVID-19 has been limited to date. This study aimed to measure the effectiveness of an automated text-based active surveillance system which was used in Cork/Kerry for the first 7 weeks of the COVID-19 response.

## Contact tracing

During the study period from 8 March to 23 April 2020 inclusive, cases were defined according to clinical criteria (presence of fever/cough/shortness of breath) and laboratory detection of severe acute respiratory syndrome coronavirus-2 (SARS-CoV-2) nucleic acid in a clinical specimen [8]. Contact tracing was undertaken for all notified cases of COVID-19 that arose in Cork/Kerry, in accordance with national protocols [3]. Contacts of confirmed cases were called individually by the Department of Public Health (DPH) for Cork/Kerry and classified as casual (< 15 min face-to-face exposure) or close (≥ 15 min face-to-face exposure). Close contacts who were symptomatic were referred for testing directly. Asymptomatic close contacts were advised about the need to self-quarantine for 14 days from the date of their last exposure to a confirmed case, and they were sent written information about their potential risk of infection with SARS-CoV-2. They were offered the option of receiving a daily text message from the DPH as part of active surveillance. Those who declined were offered the option of a daily telephone call as an alternative, but are not included in the current analysis.

## Automated text messaging

Participants’ mobile telephone numbers were added to an automated text messaging system using text broadcasting software (Saadian Technologies, Dublin, Ireland). Asymptomatic close contacts were texted every day from the day following their initial telephone call with the regional DPH until the end of their 14-day follow-up period. Text recipients were asked to provide a yes/no response to the question, *“Do you have new fever or cough or shortness of breath?”* Those who responded ‘yes’ were contacted directly by a clinician, assessed over the telephone and, if necessary, referred for priority testing for COVID-19. Those who responded ‘no’ continued with active surveillance until the end of their 14-day follow-up period. Those who responded with details of clinical queries or concerns (instead of responding ‘yes’) were contacted by a clinician or a nurse. Non-responders were sent one follow-up text after 4 h, and were then contacted directly by a clinician or a nurse if they did not respond to the second text.

## Data systems

Details of all responses to the text broadcast messaging system were exported to Microsoft Excel and collated. Of those who had been tested for COVID-19, positive results were recorded on the Computerised Infectious Disease Reporting system, Health Service Executive COVID-19 tracker, or i.Laboratory Pathology Results Enquiry system. Samples were tested in the National Virus Reference Laboratory in Dublin or in one of the regional microbiology laboratories in Cork/Kerry. Results were verified from daily line listings received from each of these laboratories.

## Ethical statement

All participants provided verbal consent during their initial telephone call with the DPH to receive a daily text message and possible contact by a clinician or nurse. They had the option to withdraw from active surveillance at any time. If requested, they were provided with relevant information pertaining to data protection legislation and compliance with the General Data Protection Regulation. In this study, we present aggregate data with no identifiable information. Thus, ethical approval was not required.

## Results

There were 1,336 asymptomatic close contacts added to the text-based active surveillance system and 12,421 texts were sent (mean: 9.3 texts per participant). The median age of respondents (or their parents/guardians) was 42 years (range: 10 months to 77 years). In total, 192 respondents (14.4%) required clinical follow-up of whom 104 (54.2%) were female and 88 (45.8%) were male. The majority (n = 120; 62.5%) were referred for testing, and the results are shown in the Table. Overall, 9.0% of close contacts were referred for testing and 2.6% tested positive for COVID-19 during follow-up.

**Table:** Results of active surveillance system for COVID-19 in Cork and Kerry, March–April 2020 (n = 1,336)

Text message recipients	n	% of tested	% of total
Required call-back by clinician (n = 1,336)			
No	1,144	NA	85.6
Yes	192	NA	14.4
Referred for testing (n = 192)			
No	72	NA	5.4
Yes	120	100	9.0
Result of testing (n = 120)			
Positive	35	29.2	2.6
Not detected	78	65.0	5.8
Invalid result	1	0.8	0.1
Not done	6	5.0	0.4

COVID-19: coronavirus disease; NA: not applicable.

Of those who required a clinical call-back, 72 (37.5%) did not meet criteria for testing; they had symptoms which were not deemed to be consistent with COVID-19, or else they sought clinical advice about returning to work, duration of self-quarantine or advice about family members or contacts. During the follow-up period, the national testing criteria for COVID-19 also changed several times as knowledge of COVID-19 and laboratory testing capacity evolved [9]. Six individuals who were referred for testing by their general practitioner (GP) were never swabbed because the eligibility criteria changed between ordering and time of testing and they no longer fit the testing criteria. One test was returned as an invalid result and the individual did not wish to be re-tested.

Overall, the response rate to daily texts was high (n = 10,300; 82.9%). Nonetheless, the absolute number of non-responses was large (n = 2,121; 17.1%) and this created a substantial workload for DPH clinical and administrative staff.

## Discussion

Active surveillance has been recommended for close contacts of other coronavirus infections such as Middle East respiratory syndrome coronavirus (MERS-CoV) [10] and SARS CoV-1 [11], but is considered too resource-intensive to be routinely recommended for other notifiable infectious diseases [12]. In the current pandemic, regional public health teams are being challenged to use their finite resources as efficiently as possible to minimise onward transmission of COVID-19. Early evidence from the COVID-19 pandemic suggests that active surveillance of close contacts does increase case detection, which in turn facilitates earlier identification of additional contacts and limits onward transmission [13]. In the first 7 weeks of the COVID-19 response in Cork/Kerry, 9.0% of close contacts who consented to participate in active surveillance were referred for testing and 2.6% tested positive for COVID-19. This is a higher detection rate than in a recent study from the United States where the positive case yield from active surveillance of 445 close contacts was 0.5% [14].

The World Health Organization has highlighted the need for robust electronic data capture tools to support efficient contact tracing and active surveillance of close contacts on a large scale [15]. Although our text message-based system resulted in the detection of additional positive cases and helped to break chains of transmission in the community, it was resource-intensive. It required manual data entry, daily data exports for follow-up and considerable input and oversight from clinical and administrative staff. In order to sustain active surveillance, extra resources are required in terms of staffing, robust IT infrastructure and strong data protection safeguards. This has also been demonstrated recently in Singapore where successful active surveillance mechanisms led to a high yield in positive cases [13]. At the time of writing, several regional public health departments in Ireland have discontinued active surveillance because of resource constraints. The system has been largely replaced by a centralised text messaging system for asymptomatic close contacts who are reminded to seek medical advice from their GP if they develop symptoms of SARS-CoV-2 infection, akin to passive surveillance.

The overall effectiveness of any active surveillance system depends on the eligibility criteria applied in testing referrals and may also involve a value judgement over what constitutes an effective yield. To our knowledge, this is the first European study to measure the positive COVID-19 yield from a text message-based active surveillance system. Older people were more inclined to opt out or request follow-up by daily telephone calls rather than by text (data not shown). There was a lack of robust data on this cohort, partly because electronic data capture tools were lacking at the outset. Further analysis of this cohort may have resulted in a greater understanding of the limitations of the text messaging system. Strict national testing criteria were in place at times because of challenges in IT infrastructure, limited laboratory capacity and large backlogs of test results with slow turnaround times owing to difficulties procuring reagents and physical swabs. These practical challenges, and the lack of testing of asymptomatic close contacts, are likely to have reduced the overall yield of positive results. Furthermore, some text recipients indicated that they did not reply to daily texts because doing so involved a cost (if using pay-as-you-go mobile telephones), and this may have impacted on the response rate. At the time of writing, Ireland has implemented testing for all symptomatic and asymptomatic close contacts of confirmed cases of COVID-19. If these criteria had applied during the study period, we may have had a higher yield of SARS-CoV-2 infections among this cohort.

Automated active surveillance systems can thus facilitate early identification of symptomatic close contacts and positive cases of COVID-19. However, it requires resourcing with robust IT infrastructure, sufficient laboratory capacity and dedicated clinical and administrative support.
